# Seronegative hepatitis C virus infection in Polish blood donors—Virological characteristics of index donations and follow‐up observations

**DOI:** 10.1002/jmv.25617

**Published:** 2019-11-21

**Authors:** Piotr Grabarczyk, Dorota Kubicka‐Russel, Aneta Kopacz, Grzegorz Liszewski, Ewa Sulkowska, Paulina Zwolińska, Kazimierz Madaliński, Maciej Marek, Małgorzata Szabelewska, Ewa Świątek, Tomasz Laskus, Marek Radkowski

**Affiliations:** ^1^ Department of Virology Institute of Haematology and Transfusion Medicine Warsaw Poland; ^2^ Department of Virology National Institute of Public Health—National Institute of Hygiene Warsaw Poland; ^3^ Labolatory of Infectious Diseases Transmitted by Blood, Regional Blood Transfusion Center Kalisz Poland; ^4^ Department of Testing for Infectious Diseases Transmitted by Transfusion Military Blood Transfusion Center Warsaw Poland; ^5^ Laboratory of Infectious Diseases Serodiagnostics, Regional Blood Transfusion Center Wrocław Poland; ^6^ Department of Adult Infectious Diseases Warsaw Medical University Warsaw Poland; ^7^ Department of Immunopathology of Infectious and Parasitic Diseases Warsaw Medical University Warsaw Poland

**Keywords:** blood donors, clinical sensitivity, EIA assays, HCV, NAT yields

## Abstract

Nucleic acid testing (NAT) was implemented in Poland in 1999 for screening of plasma for fractionation and for all blood donors in 2002. To analyze seronegative NAT‐positive samples representing hepatitis C virus (HCV) window‐period (WP) in the years 2000 to 2016 and to determine infection outcome. We analyzed results of 17 502 739 donations screened in minipools (6‐48) or individually. Index samples underwent viral load (VL) quantification, genotyping and Ag, and anti‐HCV re‐testing using chemiluminescence (CMIA), electrochemiluminescence (ECLIA), and fourth‐generation enzyme‐linked immunosorbent assay (IV EIA) assays. HCV‐seronegative infections were identified in 126 donations (7.2/mln donations; 95% confidential intervals, 6.0‐8.6). Frequency of NAT yields was decreasing over time. Of the initial 126 seronegative index cases 106 were retested: 32.1% were reactive in IV EIA, 11.3% in ECLIA, and 1.9% in CMIA. The lowest VL correlated with absent anti‐HCV and HCV Ag, while VL was highest when the antigen was detectable and then it decreased when anti‐HCV appeared at a level detectable by sensitive third generation tests while retesting. The proportion of genotype 1 was 38.9% in samples positive only for HCV RNA and 71.4% in samples that were anti‐HCV reactive in re‐testing. In parallel, genotype 3 frequency was 50% in the former group and 21% in the latter. NAT is an effective measure to limit HCV transmission by transfusion and IV EIA seems to have higher clinical sensitivity than ECLIA. Samples representing likely successive phases of early HCV infection were characterized by different genotype distribution probably due to very early elimination of genotype 3.

## Highlight

- NAT is highly effective in prevention of transmission of HCV seronegative infections.
- In nearly 7% of followed up donors antibodies appeared later than 50 days after index donation.
- The highest clinical sensitivity was demonstrated by the IVth generation tests.
- Among the third generation assays, ECLIA showed higher sensitivity than CMIA.
- Samples representing likely successive phases of early HCV infection demonstrated different genotype distribution.

## 1 BACKGROUND

Poland was one of the first countries to introduce nucleic acid amplification testing (NAT) for blood donor screening. Molecular testing was implemented in 1999 for hepatitis C virus (HCV) RNA screening of plasma for fractionation and in 2002 for all blood donation.^1^ Although all donors in Poland are nonremunerated volunteers, who undergo medical assessment and have to deny risk factors for viral infections before donation, the number of serologic window‐period (WP) donations was found to be high. Busch and colleagues reported that since the implementation of HCV NAT screening through 2008, the highest number of HCV WP infections among participating European countries was noted in Poland, with 83 out of the total 123 infections.^2^ Despite a decreasing trend, the HCV NAT‐only detection rate remains high in Poland with the residual risk of transfusion‐transmitted infection.^3^, ^4^ One recently published study aiming to explain the high rate of HCV WP donations in Poland pointed to the likely impact of questionnaire design and donor compliance.^5^

The objective of the current study was to analyze the frequency of HCV NAT yields in Poland in the years 2000 to 2016 and to provide virological characterization of index donations and follow‐up samples. Additionally, the index samples recognized in routine NAT screening (RNA HCV positive and anti‐HCV negative) were tested with different serological screening assays to evaluate their clinical sensitivity and to characterize deeper the early stage of HCV infection.

## 2 MATERIAL AND METHODS

### 2.1 Blood donations

We analyzed results of NAT testing of 17 502 739 donations collected in the years 2000 to 2016. These results were provided by 23 Blood Transfusion Centers (BTC) including 21 Regional Blood Transfusion Centers (RBTC), Military Blood Transfusion Center, and Blood Transfusion Center of The Ministry of Internal Affairs and Administration. The blood transfusion system in Poland applies uniform guidelines for the assessment of blood donor eligibility.^6^

Most donations originated from repeat blood donors (65.3%) and males (74%). Population of donors consisted mainly of young people—the majority (60%) were 18 to 30 years old (based on data available since 2005).

### 2.2 Methods of screening

NAT was performed either in minipools of plasma (6‐48 donations) or in individual donations. The minipools strategy was performed initially (2000‐2004) in minipools of 48 (MP48) with Cobas Amplicor HCV v 2.0 (Roche Molecular Systems Inc, Branchburg), in the years 2005 to 2006 it was conducted in minipools of 24 with Cobas Ampliscreen v 2.0 (Roche), and since 2007 in minipools of 6 samples—initially with Cobas Taqscreen MPX (Roche) and after 2012 with version 2.0 of Cobas Taqscreen MPX assay (Roche). Individual donation testing was performed using transcription mediated assays (TMA): initially with Procleix HCV/HIV‐1 (Gen‐Probe Incorporated, San Diego), in the years 2005 to 2009 with Procleix Ultrio (Gen‐Probe Incorporated), in the years 2010 to 2012 using Procleix Ultrio Plus (Gen‐Probe Incorporated) and since 2013 using Procleix Utrio Ellite (Gen‐Probe Incorporated). Details of clinical sensitivity of the HCV RNA assays are shown in Figure S1. BTCs which did not have their own NAT laboratory, were submitting samples for screening to another BTC. Number of laboratories conducting RNA HCV testing increased during the analyzed period from 8 to 18.

Donations were tested in parallel for anti‐HCV with one of the following immunoenzymatic assays (EIA): HCV ELISA V3.0 (Ortho‐Clinical Diagnostics, Inc a Johnson&Johnson Company, Raritan), Architect Anti‐HCV (ABBOTT, Wiesbaden, Germany), and Vitros aHCV (Ortho‐Clinical Diagnostics, Wycombe Buckinghamshire, United Kingdom) (see Figure S1).

### 2.3 Confirmatory testing

First step of confirmatory procedure was performed in screening laboratory. If minipool containing 24 to 48 donations was reactive, subpools prepared from 6 to 12 donations were tested with assay used for screening and in further step donations from reactive subpool were tested individually using the same assay.

In case of a reactive NAT result in a seronegative donation, previously unopened plasma sample from the index donation or plasma from the bag was tested for RNA HCV individually in the Institute of Hematology and Transfusion Medicine in Warsaw (IHTM). For infection confirmation in reactive donation identified in MP ≥ 24 Cobas Amplicore (95% LOD, 43 IU/mL) and later period of the analysis Cobas Ampliscreen (95% LOD, 21.4 IU/mL) assays were applied. For donations reactive in IDT and in MP6 assays characterized with 95% LOD at level of 3 IU/mL were always used (Procleix HCV/HIV‐1, Procleix Ultro, Procleix Ultrio Plus or Procleix Ultrio Elite tests). When available, follow‐up samples were tested for RNA HCV and serological markers as well.

### 2.4 Other testings

Genotype and viral load (VL) were analyzed in 106 out of 126 (84.1%) HCV RNA positive index donations for which sufficient amount of biological material was available. VL was tested in 82 donations using Cobas Amplicor HCV Monitor test v 2.0 (Roche) and in 24 donations with Confirmatory PCR Kit HCV v 1.0 (GFE Blut mbH, Frankfurt/Main, Germany) calibrated on HCV‐RNA genotype 1 reference panel P0019 (according to the leaflet to the panel; BioQControl B.V., Rijswijk, The Netherlands). According to the manufacturer limit of detection was 600 IU/mL for Roche assay and 2.9 IU/mL for GFE test. Genotype and subtype of HCV isolates were determined by Genotype Assay (LiPA) (Bayer HealthCare Tarrytown) or Versant HCV Genotype 2.0 Assay (LiPA) (Siemens Healthcare Diagnostics Inc, Tarrytown). Additionally, out of 106 available index samples 70 were tested for HCV core antigen with Architect HCV Ag assay (ABBOTT), while 36 were tested using Ortho HCV core Ag Assay (Ortho Clinical Diagnostics, Rochester).

All HCV RNA positive samples identified by NAT were analyzed with four different serological HCV screening assays: chemiluminescence assays (CMIA) Vitros aHCV (Ortho‐Clinical Diagnostics) and Architect Anti‐HCV (ABBOTT); electrochemiluminescence assay (ECLIA)—Elecsys Anti‐HCV II (Roche Diagnostics GmbH, Mannheim, Germany) and fourth‐generation enzyme‐linked immunosorbent assay (ELISA) which was also able to detect HCV core antigen (combo assay)—Monolisa HCV Ag‐Ab Ultra V2 (Bio‐Rad, Marnes‐la‐Coquette, France). In case of a reactive result, testing was repeated twice. Re‐testing was performed in plasma bags that were not accepted for clinical use due to reactive result in NAT.

### 2.5 Statistical analysis

Frequency of seronegative HCV infected donations was calculated per one million with 95% confidence intervals (95% CI). Differences between two frequencies were expressed as a relative risk with 95% CI. Differences in VL due to non‐normal distribution, were analyzed using the Kruskal‐Wallis test.

All the calculations were done using Statistica version 13 (Palo Alto, CA).

## 3 RESULTS

### 3.1 Detection of seronegative HCV infection

Altogether 126 seronegative HCV RNA positive donations were detected in the years 2000 to 2016 (7.2/1 mln donations; 95% CI, 5.9‐8.5): 50 donations were identified with Cobas Amplicor HCV v 2.0 (Roche), 14 with Cobas AmpliScreen HCV v 2.0 (Roche), 25 with CobasTaqscreen MPX (Roche), 14 with Cobas Taqscreen MPX 2.0 (Roche), 3 with Procleix HCV/HIV‐1 (Gen‐Probe), 11 with Procleix Ultrio (Gen‐Probe), 8 with Procleix Ultrio Plus (Gen‐Probe), and 1 with Procleix Utrio Ellite (Gen‐Probe).

Seventy‐four of 126 NAT yields were originally anti‐HCV negative in screening performed with HCV ELISA V3.0 (Ortho‐Clinical Diagnostics), 33 were negative when tested by Architect Anti‐HCV (ABBOTT) and the remaining 19 were negative in Vitros aHCV assay (Ortho‐Clinical Diagnostics).

**Figure 1:**
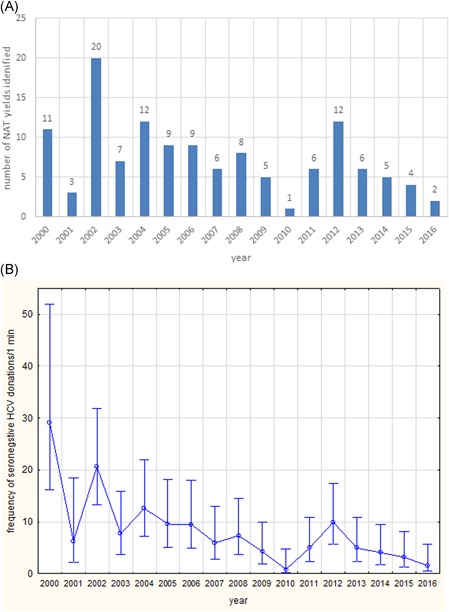
Identification of seronegative HCV infected donations in Poland, in the years 2000 to 2016: A, number of positive NAT yields in each year. B, frequency of seronegative HCV RNA positive donations expressed as a number of NAT yields per 1 mln donations with 95% confidential intervals. HCV, hepatitis C virus; NAT, nucleic acid testing

In spite of increasing analytical sensitivity of HCV RNA screening systems introduced over the years (Figure S1), we observed the highest number of NAT yields at the beginning of the study and there was a decreasing frequency of HCV RNA detection over time (Figure 1) (*P* < .05). The highest number of HCV WP infections (20 donations) was recorded in the year 2002 and the lowest (one donation) in 2010.

The frequency of seronegative HCV infections differed significantly (relative risk 2.06; 95% CI, 1.66‐3.63; *P* < .05) between first time (6.14/1 mln; 95% CI, 3.03‐9.24) and repeat (12.62/1 mln; 95% CI, 9.37‐15.87) donors; however it did not differ when the frequencies of infection in donations collected from repeat blood donors (7.32/1 mln; 95% CI, 5.86‐8.78) and first time donors (6.83/1 mln; 95% CI, 4.39‐9.28) were compared (relative risk 0.9; 95% CI, 0.6‐1.4; *P* > .05). There were no significant differences (relative risk 1.77; 95% CI, 0.95‐3.28; *P* > .05) between females (6.74/1 mln; 2.34‐9.97) and males (11.92/1mln; 8.93‐14.91). The analysis regarding first time and repeat blood donors and gender stratification was possible only since 2005, when collection of these data started.

The highest frequency of HCV infected seronegative blood donors was found among those aged 31 to 40 years (18.6/1 mln; 95% CI, 12.8‐27.0), followed by donors aged 21 to 30 (12.8/1 mln; 95% CI, 9.1‐17.9), those below 20 (6.2/1 mln; 95% CI, 3.3‐11.3), 41 to 50 (2.5/1 mln; 95% CI, 0.7‐9.0) and those 51 to 60 years old (2.7/1 mln; 95% CI, 0.5‐15.4). No seronegative infections were found among the oldest donors (>61 years old, 0/1 mln; 95% CI, 0‐120.7). Significant differences were observed between 31 and 40 years donors and group aged 21 to 30 and <20 years (*P* < .05).

Seronegative HCV infections were detected in 21 out of 23 participating BTCs: from 1 in Kraków to 15 in Białystok. No such infections were detected in Radom and in Blood Transfusion Center of The Ministry of Internal Affairs and Administration. No statistical significant differences were found between BTCs regarding the frequency of seronegative HCV‐infection detection (Figure S2).

### 3.2 Index donation characteristics

For 106 of 126 (84.1%) seronegative donations there was a sufficient amount of plasma to perform supplemental testing. Data for each individual donor are presented in Table S1.

The quantity of HCV RNA (VL) in the index samples ranged from 6 to 4.8 × 10^7^ IU/mL (mean 2.3 × 10^6^ IU/mL, median 4.4 × 10^5^ IU/mL). The distribution of genotypes was as follows: 45.3% for 3a, 40.6% for 1b, 7.5% for 4, 4.7% for 1a, and 1.9% were mixed genotypes. The majority of index donations (82.1%) were HCV Ag core positive.

**Figure 2:**
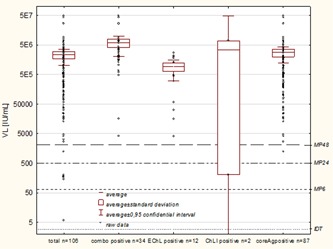
Viral load in NAT yield donations positive in IV generation anti‐HCV test (Ag/Ab combo), anti‐HCV ECLIA, and CMIA assays and in core Ag assay. On the right axis sensitivities of NAT performed in various minipools (MP) and for individual donation testing (IDT) are marked. CMIA, chemiluminescence; ECLIA, electrochemiluminescence; HCV, hepatitis C virus; NAT, nucleic acid testing

Thirty‐four (32.1%) of the 106 samples originally seronegative by third‐generation assay in screening were repeatedly reactive in the fourth‐generation ELISA and all (34/34) were tested positive in the core Ag assay. Fourteen (14/106‐13.2%) NAT yields were repeat reactive in retesting using third generation anti‐HCV tests, including 12 (11.3%) in ECLIA and another 2 (1.9%) in CMIA (Vitros). None of the donations (0/106) was reactive in the Architect test. All but one of anti‐HCV repeat reactive donations in retesting (13/14) were tested positive for HCV Ag and eight were repeatedly reactive in IV generation test. Viral characteristics of donations reactive in retesting is presented in Table 1 and Figure 2, whereas testing results of individual donations are shown in Table S1.

**Table 1:** HCV genotypes in NAT yield donations and the results of testing by IV generation anti‐HCV test (Ag/Ab combo), anti‐HCV ECLIA, CMIA, and core Ag assay

Subtype	Genotype in total	Number (%) of donations infected with HCV subtypes/genotypes among donations positive in				
		Total (%)	Combo assay (%)	ECLIA (%)	CMIA (%)	Core assay (%)
1a		5 (47.2)	2 (5.9)	2 (16.7)	0 (0)	4 (4.6)
1b		43 (40.6)	19 (55.9)	7 (58.3)	1 (50)	37 (42.5)
	**1**	**48 (45.3)**	**21 (61.8)**	**9 (75)**	**1 (50)**	**0 (0)**
3a	**3**	**48 (45.3)**	**10 (29.4)**	**2 (16.7)**	**1 (50)**	**38 (43.7)**
4		1 (0.9)	1 (2.9)	1 (8.3)	0 (0)	1 (1.1)
4a/4c/4d		1 (0.9)	0 (0)	0 (0)	0 (0)	1 (1.1)
4c/4d		6 (5.7)	0 (0)	0 (0)	0 (0)	4 (4.6)
	**4**	**8 (7.5)**	**1 (2.9)**	**1 (8.3)**	**0 (0)**	**6 (6.9)**
Mix		**2 (1.9)**	**2 (5.9)**	**0 (0)**	**0 (0)**	**2 (2.3)**
Total		**106**	**34**	**12**	**2**	**87**

Abbreviations: CMIA, chemiluminescence; ECLIA, electrochemiluminescence; HCV, hepatitis C virus; NAT, nucleic acid testing.

Numbers (%) in non‐bold refer to subtypes.

Values in bold are the sum for all subtypes belonging to genotype.

**Figure 3:**
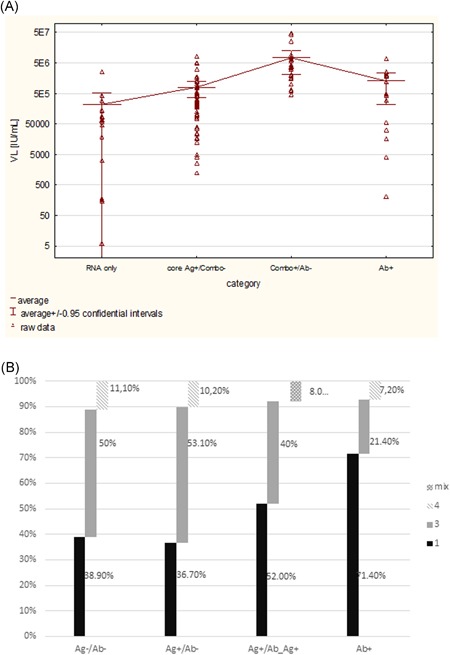
Viral characterization of four groups likely representing consecutive stages of early HCV infection: RNA HCV as a sole marker, appearance of Ag (detected in core Ag assay), combo assay positive, and anti‐HCV positive by III generation tests: A, viral load; B, genotypes (subtypes) distribution. HCV, hepatitis C virus

Based on reactivity in different assays, four distinct groups likely to represent successive stages of de novo HCV infection could be differentiated: in the first group, HCV RNA was the only infection marker, the second group was characterized by HCV RNA and HCV core antigen presence, third by anti‐HCV‐positivity when using IV generation test (Ab/Ag assay) in addition to HCV RNA and core Ag detection, and the fourth group was characterized by repeat reactivity in anti‐HCV assays and negative results in Ab/Ag and Ag assays while retesting. Eventually, 18, 49, 25, and 14 index donations represented following groups, respectively.

Significant differences in VL and genotype distribution were observed for these groups (*P* < .05) (Figure 3). In the first group (HCV RNA only), VL was relatively low, genotype 3a (50%) was predominant, while genotype 1 was less frequent (38.9%). In the second group (HCV core antigen and HCV RNA positive), both VL and genotype distributions were similar to group I. The highest concentration of viral RNA was found in donors positive for HCV RNA, core Ag and repeat reactive results in IV generation test (group III). In donations representing the last (IV) group (weak positive anti‐HCV in retesting), VL was lower as compared to donors reactive in the combo test, but negative for anti‐HCV in third‐generation assays. Moreover, compared to HCV RNA positive only donations (group I) and to samples with detectable viral RNA and Ag (group II), higher frequency of genotype 1 (71.4%) and lower percentage of genotype 3 (21.4) were observed (Figure 3).

### 3.3 Follow‐up of seronegative donors

**Figure 4:**
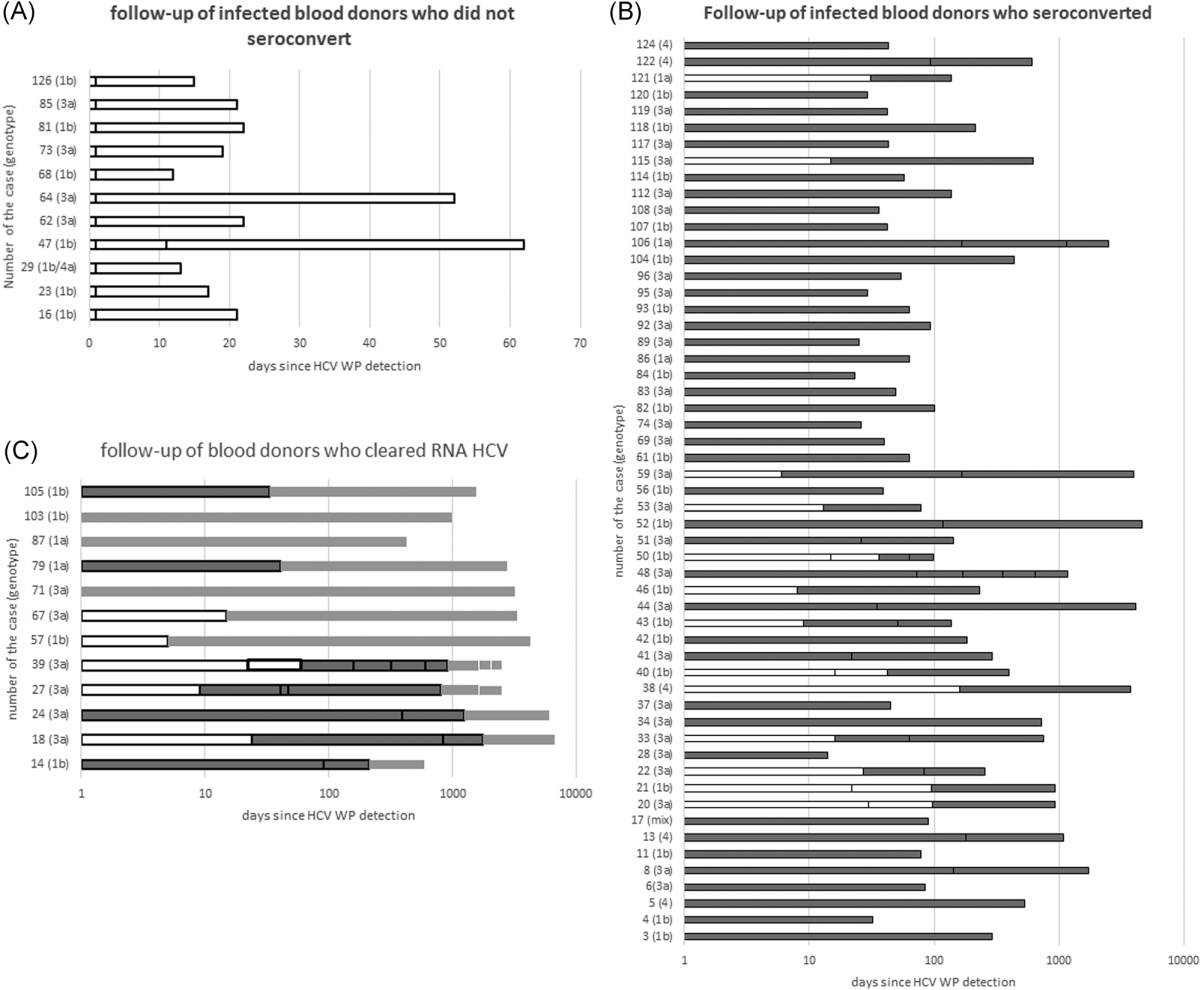
Follow‐up of infected blood donors identified in seronegative stage of HCV infection. A, No seroconversion. B, Seroconversion. C, Cleared HCV RNA. Unshaded boxes represent seronegative stage of infection, gray‐shaded boxes represent period after anti‐HCV antibodies appeared, and shaded rectangle fields without frames indicate seropositive stage after RNA HCV clearance. On graphs B and C log scale was applied for clearer presentation of the longest follow‐ups. HCV, hepatitis C virus

Follow‐up samples were available in total for 78 out of 106 (73.6%) seronegative donors: one follow‐up sample was collected from 46 donors, two consecutive samples were collected from 17, three from 10, four from two, and in three donors ≥5 samples were available. The follow‐up test results for anti‐HCV and RNA HCV are presented in Figure 4. Eleven donors remained anti‐HCV negative and HCV RNA positive representing most likely the early stage of infection (group A), 55 donors seroconverted but were still HCV RNA positive (group B) and 12 donors cleared infection (anti‐HCV positive HCV RNA‐negative; group C). Duration of follow‐up was 11 to 61 days (median 20) for group A, 13 to 4617 days (median 118) for group B and 427 to 6607 (median 2629) for group C.

For five out of 76 donors (6.6%) seroconversion was not documented in samples collected ≥50 days after the index donation. Donors 64 and 47 from group A and donors 20, 38, and 50 from group B remained seronegative in follow‐up samples collected after 51, 61, 96, 160, and 63 days, respectively. In donor 20 the second seronegative sample collected 91 days after index donation remained Western blot negative with the c100 protein reactivity only. After the next 840 days this donor was positive in serological screening assay, Western blot and remained RNA HCV positive.

Out of 14 blood donors who became RNA HCV negative, 7 were primarily infected with genotype 3a, 6 with 1b and 1 with 1a. However, four received antiviral treatment. Details on VL in the follow‐up samples are presented in Table S1.

## 4 DISCUSSION

During 17 years (2000‐2016) of HCV RNA screening in Polish blood donors, we prevented transfusion of blood components prepared from 126 donations collected at the seronegative, presumably early, stage of HCV infection. Importantly, the infectivity of seronegative donations due to their typical high viral load and the lack of even a trace amount of specific antibodies is higher compared to seropositive donations.^7^ In our study the concentration of viral RNA in seronegative donations was usually above 10^5^ IU/mL and only in 5/106 (4.7%) of identified index donations viral load was below 250 IU/mL. Moreover, in the majority (88.7%) of index samples no antibodies were detected even when retested with the later introduced serological tests. In addition, noreactivity whatsoever was observed in the Western blot test in the majority of these samples (90.6%—data not shown). While NAT testing significantly improved transfusion safety, a case of the genotype 3 infection transmission by red cells concentrate (RBC) occurred in 2004.^8^ HCV RNA concentration in the infecting sample was below the analytical sensitivity of the screening performed at that time in minipools of 48 (estimated to be about 2000 IU/mL).^8^ However, decreasing the number of donations in each MP in subsequent years improved the analytical sensitivity to at least 40 IU/mL. Applying analytical sensitivity of different screening strategies on VL data (see reference lines in the Figure 2) we assessed the probable number of missed WP HCV to be six when using MP48, five when using MP24, and one when using MP6. Theoretical calculations show that using even the most sensitive available methods cannot eliminate the risk of virus transmission completely.^7^ However, the current approach limits the diagnostic window period to about 3 days. Thus, considering the current HCV incidence among Polish donors, we can estimate the risk of transfusion‐transmitted infection to be 0.3 to 1.2 per 1 mln donations.^3^, ^9^

The majority of seronegative HCV infections were identified in the first years of our analysis, and their number significantly decreased in the following years. This downward trend probably reflects an overall improvement in the epidemiological situation of blood donors^10^ and is less likely to be influenced by the introduction of more sensitive EIAs. We observed a significant and regular decrease of seropositive (confirmed) HCV infections among first time donors from 371 (95% CI, 347‐396) in 2009 to 133 (95% CI, 115‐151) per 100 000 donors in 2016 (IHTM data). It should be noted that all our HCV NAT yield donations, which were negative in EIA 3.0 HCV Ortho, were re‐tested with the later introduced Abbott Architect assay and remained negative.

Interestingly, the highest number of seronegative infections was identified in the Podlasie region (North‐Eastern Poland), which is characterized by low frequency of seropositive HCV infections in blood donors.^5^, ^10^, ^11^ The relatively high number of infections in this region immediately after the introduction of NAT and the fact that 10 out of 14 donors were infected with genotype 3a may indicate local appearance of a group of so called “test seekers” engaged in risky behaviors.

It is also of note that only a small difference was observed in the prevalence of seronegative infection between first time and repeat time donors, which is in contrast to seropositive donors for whom this difference is much higher. In an analysis encompassing years 2005 to 2015, the relative risk for first time versus repeat blood donors was over 25 and highly significant.^10^ These observations may suggest that at least part of seronegative HCV RNA positive donations come from repeat donors who give blood to learn about their infection status. In our previous study, we reported that seronegative HCV infections may be the result of insufficient deferral criteria for risk factors including minor medical procedures, but may also result from donor noncompliance with the current criteria for donor qualification.^5^ The observed decreasing number of seronegative HCV infections over the years may also result from improved awareness of donors as to their responsibility for blood safety as well as from reduction in the number of potential sources of infection in the population due to effective and wide available antiviral treatment.^12^, ^13^

In the current analysis, we found that the distribution of genotypes in seronegative donors has not significantly changed over the years as compared to our previous report.^1^ However, this distribution differs from that observed in seropositive patients referred for antiviral treatment. Thus among our donors, who were presumably in the early stage of infection, genotype 3 predominated and almost 80% of chronic hepatitis C patients presenting for treatment are infected with genotype 1.^14^

Not unexpectedly, we found higher clinical sensitivity of IV generation test (Monolisa HCV Ag‐Ab Ultra V2, Bio‐Rad) compared to III generation assays. This observation is in line with the results of a previous multicenter study and could be explained by the fourth generation assay ability to detect viral antigen.^15^ The chance of the infection detection at the early stage can be significantly increased using Ag‐Ab assay—in our study by over 30% as compared to the primary III generation assays screening results. On the other hand the combo assay did not achieve clinical sensitivity of the test intended specifically for antigen detection, which on Polish NAT yields samples reached 82.1%. Difference in clinical sensitivity with respect to viral antigen detection between the Ag assay and combo assay suggests that the latter could be significantly improved. Ag assay is characterized by high clinical sensitivity and since it provides information on the presence and concentration of viral antigen it is being considered to be used for monitoring of treatment effectiveness and for infection confirmation in anti‐HCV repeat reactive samples.^16^, ^17^, ^18^ It is also worth noting that out of the third generation tests, ECLIA showed higher sensitivity compared to both CMIAs (*P* < .05).

Our results allowed for characterization of the early stages of HCV infection. VL differed between donors at different stage of early infection defined by distinct diagnostics markers patterns (Figure 3A). The lowest VL correlated with absent anti‐HCV and HCV Ag, while VL was highest when the antigen was detectable and then it decreased when anti‐HCV appeared at a level detectable by sensitive third generation tests while retesting.

Interestingly, changes in VL were accompanied gradual decrease in genotype 3 and increase in genotype 1 frequency. The most likely explanation is the more frequent spontaneous clearance of genotype 3, and more frequent progression of genotype 1 infection into chronicity^19^ that could also explain the domination of genotype 3a in Polish NAT yields and domination of genotype 1b among seropositive donors and patients with chronic hepatitis.^1^

Our observations suggest that the process of infection elimination could take place even before the appearance of specific antibodies and thus is likely to be dependent on cellular mechanisms. This is in line with finding of multispecific and sustained cell immune response as a key determinant of HCV clearance.^20^

In conclusion, we demonstrated high effectiveness of NAT in prevention of HCV transmission from seronegative donors. We observed a high, but decreasing over time, number of seronegative NAT yields among Polish blood donors. In nearly 7% of followed up donors antibodies appeared later than 50 days after index donation. The highest clinical sensitivity, as assessed by NAT yields testing, was demonstrated by the IV generation tests, while among the third generation assays, ECLIA showed higher sensitivity than CMIA. Samples representing successive stages of early HCV infection were characterized by differences in viral load and genotypes distribution.

## CONFLICT OF INTERESTS

The authors declare that there are no conflict of interests.

## ETHICS STATEMENT

The study was approved by Bioethical Committee at Institute of Hematology and Transfusion Medicine, Warsaw, Poland (no. 36/2016).

## Supporting information

Supplementary information
