# Supplementary material for: Seronegative hepatitis C virus infection in Polish blood donors—Virological characteristics of index donations and follow‐up observations
Source: J Med Virol. 2019 Nov 21;92(3):339–47. doi: 10.1002/jmv.25617 (PMC7003774; doi:10.1002/jmv.25617)
Supplement: Supplementary file 1 — Supplementary information [file JMV-92-339-s001.docx]

**SUPPLEMENTARY MATERIAL**

**Fig. 1S. NAT methodology used for HCV RNA screening in Polish blood donors in 2000-2016.** In the frame, at the top, EIA assays used for screening at that time are presented.

**
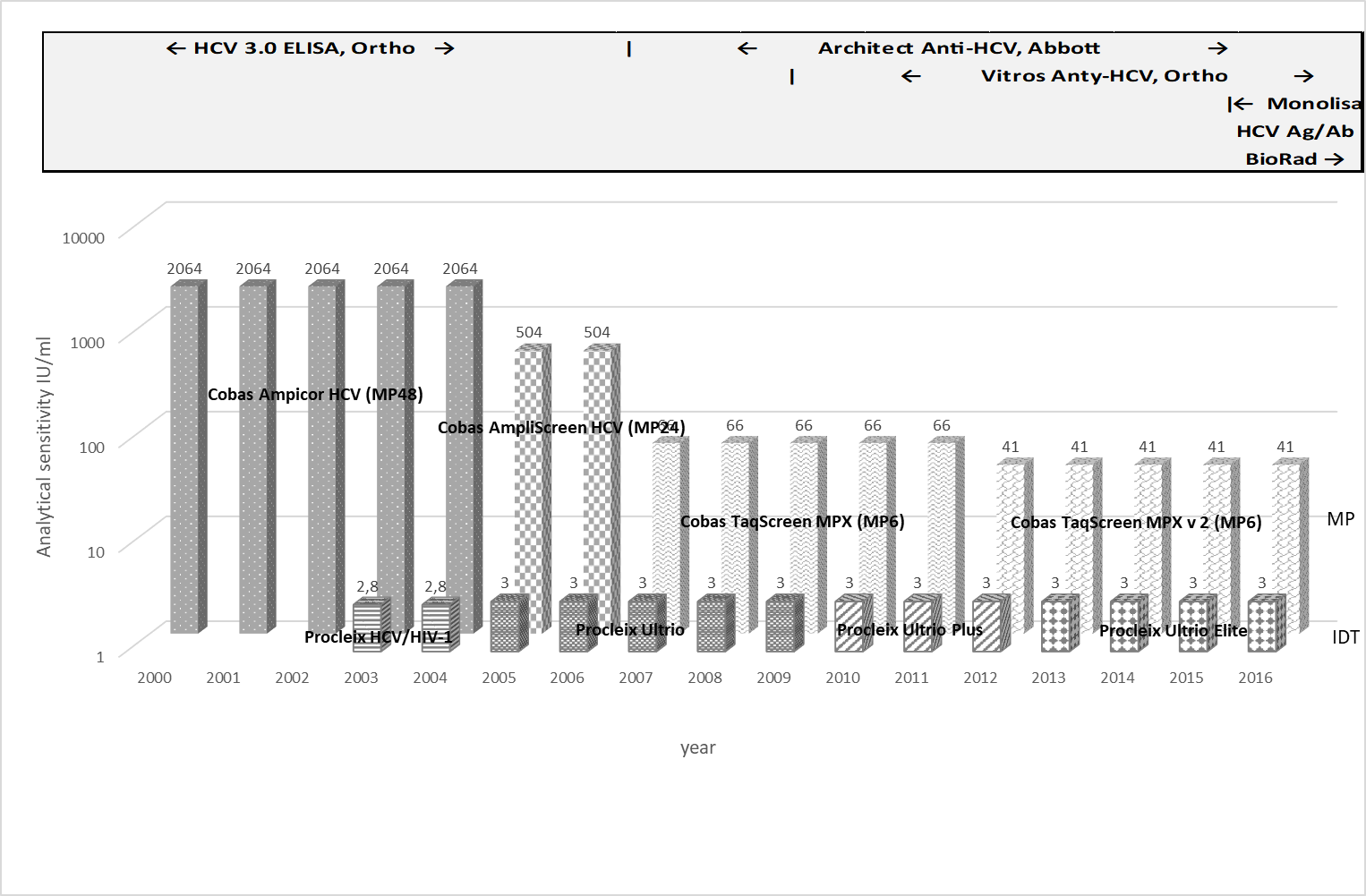
**

**Table 1S**. Virological characterization of seronegative Polish blood donors infected with HCV - testing results of index and follow-up plasma samples.

|  | **Screening tests** | | | | **viral characterization** | | |  | **results of retrospective testing** | | | |
| --- | --- | --- | --- | --- | --- | --- | --- | --- | --- | --- | --- | --- |
|  | **serological (anti-HCV)** | | **molecular (RNA HCV)** | |  |  |  | **RIBA/WB** | **with serological assays** | | | |
| **ID** | **result** | **assay** | **result** | **assay** | **HCVcAg** | **genotype** | **RNA HCV** (IU/ml) |  | **Architect** (Abbott) | **Vitros** (Ortho) | **Elecsys Anti HCV II** (Roche) | **Monolisa HCV Ag-Ab Ultra V2** (BioRad) |
| **1** | nonreactive | EIA/ELISA (Ortho) | **reactive** | Cobas Amplicor | **reactive** | 1a | 5.1E+06 | neg* | nonreactive | nonreactive | nonreactive | nonreactive |
| **2** | nonreactive | EIA/ELISA (Ortho) | **reactive** | Cobas Amplicor | **reactive** | 1b | 4.8E+06 | neg* | nonreactive | nonreactive | nonreactive | nonreactive |
| **3** | nonreactive | EIA/ELISA (Ortho) | **reactive** | Cobas Amplicor | **reactive** | 1b | 9.7E+04 | neg* | nonreactive | nonreactive | nonreactive | nonreactive |
| 3(+292) | **reactive** |  | **reactive** |  | nonreactive |  | 3.6E+05 | **pos*** |  |  |  |  |
| **4** | nonreactive | EIA/ELISA (Ortho) | **reactive** | Cobas Amplicor | **reactive** | 1b | 4.3E+07 | neg* | nonreactive | nonreactive | nonreactive | **reactive** |
| 4(+41) | **reactive** |  | **reactive** |  | **reactive** |  | 1.7E+07 | **pos*** |  |  |  |  |
| **5** | nonreactive | EIA/ELISA (Ortho) | **reactive** | Cobas Amplicor | nonreactive | 4c/4d | 5.9E+04 | neg* | nonreactive | nonreactive | nonreactive | nonreactive |
| 5(+529) | **reactive** |  | **reactive** |  | nonreactive |  | 1.1E+03 |  |  |  |  |  |
| **6** | nonreactive | EIA/ELISA (Ortho) | **reactive** | Cobas Amplicor | **reactive** | 3a | 4.2E+05 | neg* | nonreactive | nonreactive | nonreactive | nonreactive |
| 6(+84) | **reactive** |  | **reactive** |  | **reactive** |  | 7.2E+03 | **pos*** |  |  |  |  |
| **8** | nonreactive | EIA/ELISA (Ortho) | **reactive** | Cobas Amplicor | **reactive** | 3a | 3.9E+06 | neg* | nonreactive | nonreactive | nonreactive | **reactive** |
| 8(+142) | **reactive** |  | **reactive** |  | **reactive** |  | 4.1E+06 | **pos*** |  |  |  |  |
| 8(+1579) | **reactive** |  | **reactive** |  | **reactive** |  | 3.2E+06 |  |  |  |  |  |
| **9** | nonreactive | EIA/ELISA (Ortho) | **reactive** | Cobas Amplicor | **reactive** | 3a | 2.6E+07 | neg* | nonreactive | nonreactive | nonreactive | **reactive** |
| **10** | nonreactive | EIA/ELISA (Ortho) | **reactive** | Cobas Amplicor | **reactive** | 1b | 4.8E+07 | neg* | nonreactive | nonreactive | nonreactive | **reactive** |
| **11** | nonreactive | EIA/ELISA (Ortho) | **reactive** | Cobas Amplicor | nonreactive | 1b | 2.8E+05 | neg* | nonreactive | nonreactive | nonreactive | nonreactive |
| 11(+77) | **reactive** |  | **reactive** |  | nonreactive |  | 4.1E+03 | **pos*** |  |  |  |  |
| **13** | nonreactive | EIA/ELISA (Ortho) | **reactive** | Cobas Amplicor | **reactive** | 4c/4d | 1.8E+05 | neg* | nonreactive | nonreactive | nonreactive | nonreactive |
| 13(+180) | **reactive** |  | **reactive** |  | nonreactive |  | 2.5E+05 | **pos*** |  |  |  |  |
| 13(+914) | **reactive** |  | **reactive** |  |  |  |  |  |  |  |  |  |
| **14** | nonreactive | EIA/ELISA (Ortho) | **reactive** | Cobas Amplicor | nonreactive | 1b | 3.3E+03 | neg* | nonreactive | nonreactive | nonreactive | nonreactive |
| 14(+89) | **reactive** |  | **reactive** |  | nonreactive |  | 2.3E+04 | **pos*** |  |  |  |  |
| 14(+122) | **reactive** |  | **reactive** |  | nonreactive |  | 2.3E+02 | **pos*** |  |  |  |  |
| 14(+374) | **reactive** |  | nonreactive |  | nonreactive |  |  | **pos*** |  |  |  |  |
| **15** | nonreactive | EIA/ELISA (Ortho) | **reactive** | Cobas Amplicor | nonreactive | 4c/4d | 6.7E+04 | neg* | nonreactive | nonreactive | nonreactive | nonreactive |
| **16** | nonreactive | EIA/ELISA (Ortho) | **reactive** | Cobas Amplicor | **reactive** | 1b | 2.1E+06 | neg* | nonreactive | nonreactive | nonreactive | **reactive** |
| 16(+20) | nonreactive |  | **reactive** |  | reactive | 1b | 3.9E+06 | neg* |  |  |  |  |
| **17** | nonreactive | EIA/ELISA (Ortho) | **reactive** | Cobas Amplicor | **reactive** | mixed | 1.5E+06 | neg* | nonreactive | nonreactive | nonreactive | **reactive** |
| 17(+88) | **reactive** |  | **reactive** |  | reactive |  | 1,1E+06 | **pos*** |  |  |  |  |
| **18** | nonreactive | EIA/ELISA (Ortho) | **reactive** | Cobas Amplicor | nonreactive | 3a | 1.4E+05 | neg* | nonreactive | nonreactive | nonreactive | nonreactive |
| 18(+23) | nonreactive |  | **reactive** |  | nonreactive |  | 6.6E+04 | neg* |  |  |  |  |
| 18(+808) | **reactive** |  | **reactive** |  |  |  | 9.9E+04 |  |  |  |  |  |
| 18(+917) | **reactive** |  | **reactive** |  |  |  | 2.9E+04 |  |  |  |  |  |
| 18(+4859) | **reactive** | Architect (Abbott) | nonreactive | GFE Blut |  |  |  | **pos***** |  |  |  |  |
| **19** | nonreactive | EIA/ELISA (Ortho) | **reactive** | Cobas Amplicor | nonreactive | 3a | 1.4E+05 | neg* | nonreactive | nonreactive | nonreactive | nonreactive |
| **20** | nonreactive | EIA/ELISA (Ortho) | **reactive** | Cobas Amplicor | **reactive** | 3a | 6.6E+05 | neg* | nonreactive | nonreactive | nonreactive | **reactive** |
| 20(+29) | nonreactive |  | **reactive** |  | reactive |  | 6.6E+05 | neg* |  |  |  |  |
| 20(+67) | nonreactive |  | **reactive** |  | reactive |  | 3.6E+05 | neg* |  |  |  |  |
| 20(+840) | **reactive** |  | **reactive** |  |  |  | 8.6E+05 | **pos*** |  |  |  |  |
| **21** | nonreactive | EIA/ELISA (Ortho) | **reactive** | Cobas Amplicor | **reactive** | 1b | 1.8E+06 | neg* | nonreactive | nonreactive | **reactive** | **reactive** |
| 21(+21) | **reactive** |  | **reactive** |  | **reactive** |  | 4.6E+06 | ind* |  |  |  |  |
| 21(+74) | **reactive** |  | **reactive** |  | nonreactive |  | 1.6E+02 | **pos*** |  |  |  |  |
| 21(+840) | **reactive** |  | nonreactive |  |  |  |  |  |  |  |  |  |
| **22** | nonreactive | EIA/ELISA (Ortho) | **reactive** | Cobas Amplicor | nonreactive | 3a | 4.6E+04 | neg* | nonreactive | nonreactive | nonreactive | nonreactive |
| 22(+26) | nonreactive |  | **reactive** |  | reactive |  | 2.4E+04 | ind* |  |  |  |  |
| 22(+56) | **reactive** |  | **reactive** |  | reactive |  | 6.0E+05 | **pos*** |  |  |  |  |
| 22(+171) | **reactive** |  | **reactive** |  | reactive |  | 3.2E+02 | **pos*** |  |  |  |  |
| **23** | nonreactive | EIA/ELISA (Ortho) | **reactive** | Cobas Amplicor | **reactive** | 1b | 1.6E+06 | neg* | nonreactive | nonreactive | nonreactive | **reactive** |
| 23(+16) | nonreactive |  | **reactive** |  | reactive |  | 7.6E+05 | ind* |  |  |  |  |
| **24** | nonreactive | EIA/ELISA (Ortho) | **reactive** | Cobas Amplicor | nonreactive | 3a | 8.2E+04 | neg* | nonreactive | nonreactive | nonreactive | nonreactive |
| 24(+390) | **reactive** |  | **reactive** |  | nonreactive |  | 1.5E+05 | **pos*** |  |  |  |  |
| 24(+848) | **reactive** |  | **reactive** |  |  |  | 7.5E+04 |  |  |  |  |  |
| 24(+4839) | **reactive** | Architect (Abbott) | nonreactive^ | GFE Blut |  |  |  | **pos***** |  |  |  |  |
| **26** | nonreactive | EIA/ELISA (Ortho) | **reactive** | Cobas Amplicor | **reactive** | 3a | 3.0E+06 | neg* | nonreactive | nonreactive | nonreactive | **reactive** |
| **27** | nonreactive | EIA/ELISA (Ortho) | **reactive** | Cobas Amplicor | nonreactive | 3a | 9.0E+04 | neg* | nonreactive | nonreactive | nonreactive | nonreactive |
| 27(+8) | nonreactive |  | **reactive** |  | reactive |  | 3.3E+05 | neg* |  |  |  |  |
| 27(+32) | **reactive** |  | **reactive** |  | reactive |  | 3.7E+05 | ind* |  |  |  |  |
| 27(+65) | **reactive** |  | **reactive** |  | reactive |  | 5.6E+04 | ind* |  |  |  |  |
| 27(+750) | **reactive** |  | **reactive** |  |  |  | 9.5E+05 | **pos*** |  |  |  |  |
| 27(+844) | **reactive** |  | nonreactive^ |  |  |  |  |  |  |  |  |  |
| 27(+878) | **reactive** |  | nonreactive^ |  |  |  |  |  |  |  |  |  |
| **28** | nonreactive | EIA/ELISA (Ortho) | **reactive** | Cobas Amplicor | **reactive** | 3a | 5.5E+05 | neg* | nonreactive | nonreactive | nonreactive | nonreactive |
| 28(+13) | **reactive** |  | **reactive** |  | reactive |  | 1.2E+05 | neg* |  |  |  |  |
| **29** | nonreactive | EIA/ELISA (Ortho) | **reactive** | Cobas Amplicor | **reactive** | mixed | 9.2E+06 | neg* | nonreactive | nonreactive | nonreactive | **reactive** |
| 29(+12) | nonreactive |  | **reactive** |  | reactive |  | 5.1E+06 | neg* |  |  |  |  |
| **30** | nonreactive | EIA/ELISA (Ortho) | **reactive** | Cobas Amplicor | **reactive** | 4c/4d | 2.0E+05 | neg* | nonreactive | nonreactive | nonreactive | nonreactive |
| **31** | nonreactive | EIA/ELISA (Ortho) | **reactive** | Cobas Amplicor | **reactive** | 3a | 1.0E+05 | neg* | nonreactive | nonreactive | nonreactive | nonreactive |
| **32** | nonreactive | EIA/ELISA (Ortho) | **reactive** | Cobas Amplicor | **reactive** | 1b | 3.0E+05 | neg* | nonreactive | nonreactive | **reactive** | nonreactive |
| **33** | nonreactive | EIA/ELISA (Ortho) | **reactive** | Cobas Amplicor | nonreactive | 3a | 6.9E+04 | neg* | nonreactive | nonreactive | nonreactive | nonreactive |
| 33(+15) | nonreactive |  | **reactive** |  | reactive |  | 4.6E+05 | neg* |  |  |  |  |
| 33(+47) | **reactive** |  | **reactive** |  | reactive |  | 2.5E+05 | neg* |  |  |  |  |
| 33(+690) | **reactive** |  | **reactive** |  |  |  | 1.5E+06 | **pos*** |  |  |  |  |
| **34** | nonreactive | EIA/ELISA (Ortho) | **reactive** | Cobas Amplicor | **reactive** | 3a | 8.0E+06 | neg* | nonreactive | nonreactive | nonreactive | nonreactive |
| 34(+720) | **reactive** |  | **reactive** |  |  |  | 4.2E+06 |  |  |  |  |  |
| **36** | nonreactive | EIA/ELISA (Ortho) | **reactive** | Cobas Amplicor | **reactive** | 3a | 3.7E+06 | neg** | nonreactive | nonreactive | nonreactive | **reactive** |
| **37** | nonreactive | EIA/ELISA (Ortho) | **reactive** | Cobas Amplicor | nonreactive | 3a | 4.4E+05 | neg* | nonreactive | nonreactive | nonreactive | nonreactive |
| 37(+44) | **reactive** |  | **reactive** |  | nonreactive |  | 1.4E+05 | neg* |  |  |  |  |
| **38** | nonreactive | EIA/ELISA (Ortho) | **reactive** | Cobas Amplicor | **reactive** | 4c/4d | 3.0E+05 | neg* | nonreactive | nonreactive | nonreactive | nonreactive |
| 38(+160) | nonreactive |  | nt |  |  |  |  |  |  |  |  |  |
| 38(+3552) | **reactive** | Architect (Abbott) | **reactive** | GFE Blut |  |  | 1.2E+05 | **pos**** |  |  |  |  |
| **39** | nonreactive | EIA/ELISA (Ortho) | **reactive** | Cobas Amplicor | **reactive** | 1b | 5.7E+05 | neg* | nonreactive | nonreactive | nonreactive | nonreactive |
| 39(+21) | nonreactive |  | **reactive** |  | reactive |  | 2.6E+05 | neg* |  |  |  |  |
| 39(+38) | **reactive** |  | **reactive** |  | nonreactive |  | 6.3E+03 | **pos*** |  |  |  |  |
| 39(+98) | **reactive** |  | **reactive** |  | nonreactive |  | 5.6E+02 | **pos*** |  |  |  |  |
| 39(+162) | **reactive** |  | **reactive** |  | nonreactive |  | 6.6E+02 |  |  |  |  |  |
| 39(+276) | **reactive** |  | **reactive** |  |  |  | 4.2E+05 |  |  |  |  |  |
| 39(+319) | **reactive** |  | **reactive** |  |  |  | 9.2E+06 |  |  |  |  |  |
| 39(+333) | **reactive** |  | **reactive** |  |  |  | 7.0E+04 |  |  |  |  |  |
| 39(+394) | **reactive** |  | nonreactive |  |  |  |  |  |  |  |  |  |
| 39(+420) | **reactive** |  | nonreactive |  |  |  |  |  |  |  |  |  |
| 39(+446) | **reactive** |  | nonreactive |  |  |  |  |  |  |  |  |  |
| **40** | nonreactive | EIA/ELISA (Ortho) | **reactive** | Cobas Amplicor | nonreactive | 1b | 1.9E+04 | neg* | nonreactive | nonreactive | nonreactive | nonreactive |
| 40(+15) | nonreactive |  | **reactive** |  | nonreactive |  | 8.7E+04 | neg* |  |  |  |  |
| 40(+26) | nonreactive |  | **reactive** |  | nonreactive |  | 1.0E+05 | neg* |  |  |  |  |
| 40(+358) | **reactive** |  | **reactive** |  |  |  | 9.2E+04 | **pos*** |  |  |  |  |
| **41** | nonreactive | EIA/ELISA (Ortho) | **reactive** | Cobas Amplicor | **reactive** | 3a | 5.7E+04 | neg* | nonreactive | nonreactive | **reactive** | nonreactive |
| 41(+21) | **reactive** |  | **reactive** |  |  |  | 3.5E+04 |  |  |  |  |  |
| 41(+271) | **reactive** |  | **reactive** |  |  |  | 3.2E+04 |  |  |  |  |  |
| **42** | nonreactive | EIA/ELISA (Ortho) | **reactive** | Cobas Amplicor | **reactive** | 1b | 4.4E+05 | neg* | nonreactive | nonreactive | **reactive** | nonreactive |
| 42(+184) | **reactive** |  | **reactive** |  |  |  | 6.7E+03 | **pos*** |  |  |  |  |
| **43** | nonreactive | EIA/ELISA (Ortho) | **reactive** | Cobas Amplicor | **reactive** | 1b | 6.7E+06 | neg* | nonreactive | nonreactive | nonreactive | **reactive** |
| 43(+8) | nonreactive |  | **reactive** |  | reactive |  | 7.2E+06 | neg* |  |  |  |  |
| 43(+42) | **reactive** |  | **reactive** |  | reactive |  | 1.4E+06 | **pos*** |  |  |  |  |
| 43(+86) | **reactive** |  | **reactive** |  |  |  | 4.6E+02 |  |  |  |  |  |
| **44** | nonreactive | EIA/ELISA (Ortho) | **reactive** | Cobas Amplicor | **reactive** | 3a | 3.6E+04 | neg* | nonreactive | nonreactive | nonreactive | nonreactive |
| 44(+34) | **reactive** |  | **reactive** |  |  |  | 3.9E+04 | ind* |  |  |  |  |
| 44(+4117) | **reactive** | Architect (Abbott) | nonreactive^ | GFE Blut |  |  |  | **pos***** |  |  |  |  |
| **45** | nonreactive | EIA/ELISA (Ortho) | **reactive** | Cobas Amplicor | **reactive** | 3a | 1.9E+06 | neg* | nonreactive | nonreactive | nonreactive | **reactive** |
| **46** | nonreactive | EIA/ELISA (Ortho) | **reactive** | Procleix HCV/HIV-1 | **reactive** | 1b | 7.7E+05 | neg* | nonreactive | nonreactive | nonreactive | nonreactive |
| 46(+7) | nonreactive |  | **reactive** |  |  |  | 5.7E+05 | neg* |  |  |  |  |
| 46(+223) | **reactive** |  | **reactive** |  |  |  | 6.0E+02 | **pos*** |  |  |  |  |
| **47** | nonreactive | EIA/ELISA (Ortho) | **reactive** | Cobas Amplicor | **reactive** | 1b | 3.5E+06 | neg* | nonreactive | nonreactive | nonreactive | **reactive** |
| 47(+10) | nonreactive |  | **reactive** |  | reactive |  | 4.2E+06 | neg* |  |  |  |  |
| 47(+51) | nonreactive |  |  |  |  |  |  |  |  |  |  |  |
| **48** | nonreactive | EIA/ELISA (Ortho) | **reactive** | Cobas Amplicor | **reactive** | 3a | 1.8E+06 | neg* | nonreactive | nonreactive | nonreactive | nonreactive |
| 48(+71) | **reactive** |  | **reactive** |  | reactive |  | 2.5E+03 | **pos*** |  |  |  |  |
| 48(+97) | **reactive** |  | **reactive** |  |  |  | 4.6E+04 | **pos*** |  |  |  |  |
| 48(+187) | **reactive** |  | nonreactive |  |  |  |  |  |  |  |  |  |
| 48(+288) | **reactive** |  | nonreactive |  |  |  |  |  |  |  |  |  |
| 48(+518) | **reactive** |  | **reactive** |  |  |  | 7.9E+03 |  |  |  |  |  |
| **49** | nonreactive | EIA/ELISA (Ortho) | **reactive** | Cobas Amplicor | **reactive** | 3a | 1.3E+06 | neg* | nonreactive | nonreactive | nonreactive | nonreactive |
| **50** | nonreactive | EIA/ELISA (Ortho) | **reactive** | Procleix HCV/HIV-1 | **reactive** | 1b | 8.1E+05 | neg* | nonreactive | nonreactive | nonreactive | nonreactive |
| 50(+14) | nonreactive |  | **reactive** |  |  |  | 3.1E+06 | neg* |  |  |  |  |
| 50(+21) | nonreactive |  | **reactive** |  |  |  | 4.6E+06 | neg* |  |  |  |  |
| 50(+28) | nonreactive |  | **reactive** |  |  |  | 1.5E+07 | neg* |  |  |  |  |
| 50(+35) | **reactive** |  | **reactive** |  |  |  | 6.9E+06 | **pos*** |  |  |  |  |
| **51** | nonreactive | EIA/ELISA (Ortho) | **reactive** | cobas Amplicor | **reactive** | 3a | 4.0E+05 | neg* | nonreactive | nonreactive | nonreactive | nonreactive |
| 51(+25) | **reactive** |  | **reactive** |  |  |  | 8.6E+02 | neg* |  |  |  |  |
| 51(+116) | **reactive** |  | **reactive** |  |  |  | 1.9E+04 | **pos*** |  |  |  |  |
| **52** | nonreactive | EIA/ELISA (Ortho) | **reactive** | Procleix HCV/HIV-1 | **reactive** | 1b | 7.8E+06 | neg* | nonreactive | nonreactive | nonreactive | **reactive** |
| 52(+116) | **reactive** |  | **reactive** |  |  |  | 1.6E+05 | **pos*** |  |  |  |  |
| 52(+4501) | **reactive** | Architect (Abbott) | **reactive** | GFE Blut |  |  | 4.7E+04 | **pos***** |  |  |  |  |
| **53** | nonreactive | EIA/ELISA (Ortho) | **reactive** | Cobas Ampliscreen | **reactive** | 3a | 1.9E+05 | neg* | nonreactive | nonreactive | nonreactive | nonreactive |
| 53(+12) | nonreactive |  | **reactive** |  |  |  | 4.3E+04 | neg* |  |  |  |  |
| 53(+65) | **reactive** |  | **reactive** |  |  |  | 1.6E+04 | **pos*** |  |  |  |  |
| **56** | nonreactive | EIA/ELISA (Ortho) | **reactive** | Cobas Ampliscreen | **reactive** | 1b | 6.9E+06 | neg* | nonreactive | **reactive** | nonreactive | **reactive** |
| 56(+38) | **reactive** |  | **reactive** |  |  |  | 3.4E+02 | **pos*** |  |  |  |  |
| **57** | nonreactive | EIA/ELISA (Ortho) | **reactive** | Cobas Ampliscreen | **reactive** | 1b | 8.7E+05 | neg* | nonreactive | nonreactive | nonreactive | nonreactive |
| 57(+4) | nonreactive |  | **reactive** |  |  |  | 9.4E+05 | neg* |  |  |  |  |
| 57(+4250) | **reactive** | Architect (Abbott) | nonreactive | GFE Blut |  |  |  | **pos***** |  |  |  |  |
| **58** | nonreactive | EIA/ELISA (Ortho) | **reactive** | Cobas Ampliscreen | **reactive** | 1b | 4.3E+06 | neg* | nonreactive | nonreactive | nonreactive | **reactive** |
| **59** | nonreactive | EIA/ELISA (Ortho) | **reactive** | Cobas Ampliscreen | **reactive** | 3a | 9.8E+05 | neg* | nonreactive | nonreactive | nonreactive | nonreactive |
| 59(+5) | nonreactive |  | **reactive** |  |  |  | 8.5E+05 | neg* |  |  |  |  |
| 59(+159) | **reactive** | Architect (Abbott) | **reactive** |  |  |  | 2.7E+04 |  |  |  |  |  |
| 59(+3832) | **reactive** | Architect (Abbott) | **reactive** | GFE Blut |  |  | 9.0E+04 | **pos***** |  |  |  |  |
| **60** | nonreactive | EIA/ELISA (Ortho) | **reactive** | Cobas Ampliscreen | **reactive** | 1b | 4.4E+05 | neg** | nonreactive | nonreactive | nonreactive | nonreactive |
| **61** | nonreactive | EIA/ELISA (Ortho) | **reactive** | Procleix Ultrio | **reactive** | 1b | 1.3E+04 | neg** | nonreactive | nonreactive | nonreactive | nonreactive |
| 61(+52) | **reactive** |  | **reactive** |  |  |  | 4.9E+05 |  |  |  |  |  |
| **62** | nonreactive | EIA/ELISA (Ortho) | **reactive** | Cobas Ampliscreen | **reactive** | 3a | 1.8E+04 | neg** | nonreactive | nonreactive | nonreactive | nonreactive |
| 62(+21) | nonreactive |  | **reactive** |  |  |  | 2.6E+04 |  |  |  |  |  |
| **63** | nonreactive | EIA/ELISA (Ortho) | **reactive** | Procleix Ultrio | **reactive** | 1b | 7.8E+05 | neg** | nonreactive | nonreactive | nonreactive | nonreactive |
| **64** | nonreactive | EIA/ELISA (Ortho) | **reactive** | Procleix Ultrio | **reactive** | 3a | 2.8E+05 | neg** | nonreactive | nonreactive | nonreactive | nonreactive |
| 64(+51) | nonreactive |  | **reactive** |  |  |  | 1.1E+05 |  |  |  |  |  |
| **66** | nonreactive | EIA/ELISA (Ortho) | **reactive** | Procleix Ultrio | **reactive** | 3a | 1.5E+06 | neg** | nonreactive | nonreactive | nonreactive | **reactive** |
| **67** | nonreactive | EIA/ELISA (Ortho) | **reactive** | Cobas Ampliscreen | **reactive** | 3a | 3.1E+06 | neg** | nonreactive | nonreactive | nonreactive | **reactive** |
| 67(+14) | nonreactive |  | **reactive** |  |  |  | 2.6E+06 |  |  |  |  |  |
| 67(+3323) | **reactive** | Architect (Abbott) | nonreactive | GFE Blut |  |  |  | **pos***** |  |  |  |  |
| **68** | nonreactive | EIA/ELISA (Ortho) | **reactive** | Cobas Ampliscreen | **reactive** | 1b | 1.0E+06 | neg** | nonreactive | nonreactive | nonreactive | **reactive** |
| 68(+11) | nonreactive |  | **reactive** |  |  |  | 5.2E+05 |  |  |  |  |  |
| **69** | nonreactive | EIA/ELISA (Ortho) | **reactive** | Cobas Ampliscreen | **reactive** | 3a | 1.3E+06 | neg** | nonreactive | nonreactive | nonreactive | nonreactive |
| 69(+39) | **reactive** |  | **reactive** |  |  |  | 5.3E+05 |  |  |  |  |  |
| **70** | nonreactive | EIA/ELISA (Ortho) | **reactive** | Cobas Ampliscreen | **reactive** | 4c/4d | 2.5E+05 | neg** | nonreactive | nonreactive | nonreactive | nonreactive |
| **71** | nonreactive | Architect (Abbott) | **reactive** | Taqscreen MPX | **reactive** | 3a | 7.3E+04 | **pos**** | ST | nonreactive | nonreactive | nonreactive |
| 71(+3177) | nonreactive | Architect (Abbott) | nonreactive^ | GFE Blut |  |  |  | neg*** |  |  |  |  |
| **73** | nonreactive | Architect (Abbott) | **reactive** | Taqscreen MPX | **reactive** | 3a | 2.3E+05 | neg** | ST | nonreactive | nonreactive | nonreactive |
| 73(+18) | nonreactive |  | **reactive** |  |  |  |  |  |  |  |  |  |
| **74** | nonreactive | Architect (Abbott) | **reactive** | Procleix Ultrio | **reactive** | 3a | 5.9E+06 | neg** | ST | nonreactive | nonreactive | **reactive** |
| 74(+25) | **reactive** |  | **reactive** |  |  |  |  |  |  |  |  |  |
| **76** | nonreactive | Architect (Abbott) | **reactive** | Procleix Ultrio | **reactive** | 1b | 6.7E+05 | neg** | ST | nonreactive | nonreactive | **reactive** |
| **78** | nonreactive | Architect (Abbott) | **reactive** | Taqscreen MPX | **reactive** | 3a | 1.8E+05 | neg** | ST | nonreactive | nonreactive | nonreactive |
| **79** | nonreactive | Architect (Abbott) | **reactive** | Procleix Ultrio | **reactive** | 1a | 4.7E+05 | neg** | ST | nonreactive | **reactive** | **reactive** |
| 79(+40) | **reactive** |  | **reactive** |  |  |  |  |  |  |  |  |  |
| 79(+2701) | **reactive** | Architect (Abbott) | nonreactive | GFE Blut |  |  |  | **pos***** |  |  |  |  |
| **80** | nonreactive | Architect (Abbott) | **reactive** | Taqscreen MPX | nonreactive | 1b | 6.0E+00 | neg** | ST | nonreactive | nonreactive | nonreactive |
| **81** | nonreactive | Architect (Abbott) | **reactive** | Procleix Ultrio | nonreactive | 1b | 1.8E+02 | neg** | ST | nonreactive | nonreactive | nonreactive |
| 81(+21) | nonreactive |  | **reactive** |  |  |  |  |  |  |  |  |  |
| **82** | nonreactive | Architect (Abbott) | **reactive** | Taqscreen MPX | **reactive** | 1b | 2.0E+06 | neg** | ST | nonreactive | nonreactive | **reactive** |
| 82(+100) | **reactive** |  | **reactive** |  |  |  |  |  |  |  |  |  |
| **83** | nonreactive | Architect (Abbott) | **reactive** | Taqscreen MPX | **reactive** | 3a | 2.7E+03 | **pos*** | ST | nonreactive | nonreactive | nonreactive |
| 83(+48) | **reactive** |  | **reactive** |  |  |  |  |  |  |  |  |  |
| **84** | nonreactive | Architect (Abbott) | **reactive** | Taqscreen MPX | **reactive** | 1b | 1.3E+06 | neg** | ST | nonreactive | **reactive** | **reactive** |
| 84(+22) | **reactive** |  | **reactive** |  |  |  |  |  |  |  |  |  |
| **85** | nonreactive | Architect (Abbott) | **reactive** | Taqscreen MPX | nonreactive | 3a | 1.6E+02 | neg** | ST | nonreactive | nonreactive | nonreactive |
| 85(+20) | nonreactive |  | **reactive** |  |  |  |  |  |  |  |  |  |
| **86** | nonreactive | Architect (Abbott) | **reactive** | Taqscreen MPX | **reactive** | 1a | 8.1E+04 | neg** | ST | nonreactive | nonreactive | nonreactive |
| 86(+62) | **reactive** |  | **reactive** |  |  |  |  |  |  |  |  |  |
| **87** | nonreactive | Architect (Abbott) | **reactive** | Procleix Ultrio | **reactive** | 3a | 1.6E+06 | neg** | ST | nonreactive | nonreactive | **reactive** |
| 87(+427) | **reactive** |  | nonreactive |  |  |  |  |  |  |  |  |  |
| **89** | nonreactive | Architect (Abbott) | **reactive** | Taqscreen MPX | nonreactive | 3a | 2.5E+06 | neg** | ST | nonreactive | nonreactive | nonreactive |
| 89(+24) | **reactive** |  | **reactive** |  |  |  |  |  |  |  |  |  |
| **90** | nonreactive | Vitros (Ortho) | **reactive** | Taqscreen MPX | **reactive** | 1b | 2.1E+05 | neg** | nonreactive | ST | nonreactive | nonreactive |
| **91** | nonreactive | Vitros (Ortho) | **reactive** | Taqscreen MPX | **reactive** | 1b | 4.8E+05 | neg** | nonreactive | ST | nonreactive | nonreactive |
| **92** | nonreactive | Vitros (Ortho) | **reactive** | Taqscreen MPX | **reactive** | 3a | 2.3E+04 | neg** | nonreactive | ST | nonreactive | nonreactive |
| 92(+92) | **reactive** |  | **reactive** |  |  |  |  |  |  |  |  |  |
| **93** | nonreactive | Architect (Abbott) | **reactive** | Taqscreen MPX | **reactive** | 1b | 1.3E+05 | neg** | ST | nonreactive | nonreactive | nonreactive |
| 93(+62) | **reactive** |  | **reactive** |  |  |  | 3.0E+06 |  |  |  |  |  |
| **95** | nonreactive | Vitros (Ortho) | **reactive** | Taqscreen MPX | **reactive** | 3a | 1.2E+03 | neg** | nonreactive | ST | nonreactive | nonreactive |
| 95(+28) | **reactive** |  | **reactive** |  |  |  | 4.5E+02 | **pos***** |  |  |  |  |
| **96** | nonreactive | Architect (Abbott) | **reactive** | Procleix Ultrio Plus | **reactive** | 3a | 4.0E+04 | neg** | ST | nonreactive | nonreactive | nonreactive |
| 96(+53) | **reactive** |  | **reactive** |  |  |  |  | ind* |  |  |  |  |
| **98** | nonreactive | Vitros (Ortho) | **reactive** | Taqscreen MPX | nonreactive | 1b | 1.8E+02 | neg** | nonreactive | ST | nonreactive | nonreactive |
| **99** | nonreactive | Vitros (Ortho) | **reactive** | Taqscreen MPX | **reactive** | 3a | 4.8E+03 | neg** | nonreactive | ST | nonreactive | nonreactive |
| **103** | nonreactive | Architect (Abbott) | **reactive** | Procleix Ultrio Plus | **reactive** | 1b | 2.2E+06 | **pos**** | ST | nonreactive | **reactive** | **reactive** |
| 103(+984) | **reactive** | Architect (Abbott) | nonreactive | GFE Blut |  |  | 2.7E+05 | **pos***** |  |  |  |  |
| **104** | nonreactive | Vitros (Ortho) | **reactive** | Taqscreen MPX | **reactive** | 1b | 4.1E+03 | neg** | nonreactive | ST | nonreactive | nonreactive |
| 104(+433) | **reactive** |  | **reactive** | GFE Blut |  |  | 2.7E+05 | pos*** |  |  |  |  |
| **105** | nonreactive | Vitros (Ortho) | **reactive** | Taqscreen MPX | **reactive** | 1b | 4.7E+05 | neg** | nonreactive | ST | nonreactive | **reactive** |
| 105(+32) | **reactive** |  |  |  |  |  |  |  |  |  |  |  |
| 105(+1509) | **reactive** | Architect (Abbott) | nonreactive | GFE Blut |  |  |  | ind*** |  |  |  |  |
| **106** | nonreactive | Vitros (Ortho) | **reactive** | Taqscreen MPX | **reactive** | 1a | 4.2E+03 | neg** | nonreactive | ST | **reactive** | **reactive** |
| 106(+164) | **reactive** |  | **reactive** |  |  |  |  |  |  |  |  |  |
| 106(+975) | **reactive** | Architect (Abbott) | **reactive** | GFE Blut |  |  | 1.6E+05 | **pos***** |  |  |  |  |
| 106(+1356) | **reactive** | Architect (Abbott) | **reactive** | GFE Blut |  |  | 1.7E+05 |  |  |  |  |  |
| **107** | nonreactive | Architect (Abbott) | **reactive** | Procleix Ultrio Plus | **reactive** | 1b | 4.0E+04 | neg** | ST | nonreactive | nonreactive | nonreactive |
| 107(+41) | **reactive** |  | **reactive** |  |  |  |  |  |  |  |  |  |
| **108** | nonreactive | Architect (Abbott) | **reactive** | Taqscreen MPX 2.0 | nonreactive | 3a | 2.1E+02 | neg*** | ST | **reactive** | nonreactive | nonreactive |
| 108(+35) | **reactive** |  | **reactive** | GFE Blut |  |  | 4.3E+03 |  |  |  |  |  |
| **110** | nonreactive | EIA/ELISA (Ortho) | **reactive** | Procleix Ultrio Plus | **reactive** | 1b | 6.0E+05 | neg*** | nonreactive | nonreactive | nonreactive | **reactive** |
| **111** | nonreactive | Vitros (Ortho) | **reactive** | Taqscreen MPX | **reactive** | 3a | 5.3E+05 | neg*** | nonreactive | ST | nonreactive | nonreactive |
| **112** | nonreactive | Vitros (Ortho) | **reactive** | Taqscreen MPX 2.0 | **reactive** | 3a | 1.6E+06 | neg*** | nonreactive | ST | nonreactive | nonreactive |
| 112(+137) | **reactive** |  | **reactive** | GFE Blut |  |  | 3.4E+04 | **pos***** |  |  |  |  |
| **114** | nonreactive | EIA/ELISA (Ortho) | **reactive** | Procleix Ultrio Elite | **reactive** | 1b | 2.6E+06 | neg*** | nonreactive | nonreactive | **reactive** | **reactive** |
| 114(+57) | **reactive** |  | **reactive** | GFE Blut |  |  | 1,0E+01 | **pos***** |  |  |  |  |
| **115** | nonreactive | Vitros (Ortho) | **reactive** | Taqscreen MPX 2.0 | **reactive** | 3a | 1.6E+04 | neg*** | nonreactive | ST | nonreactive | nonreactive |
| 115(+14) | nonreactive |  | **reactive** | GFE Blut |  |  | 1.4E+04 | neg*** |  |  |  |  |
| 115(+601) | **reactive** | Architect (Abbott) | **reactive** | GFE Blut |  |  | 3,0E+05 | ind*** |  |  |  |  |
| **117** | nonreactive | Architect (Abbott) | **reactive** | Taqscreen MPX 2.0 | **reactive** | 3a | 1.3E+06 | neg*** | ST | nonreactive | nonreactive | nonreactive |
| 117(+42) | **reactive** |  | **reactive** | GFE Blut |  |  | 1.5E+04 | neg*** |  |  |  |  |
| **118** | nonreactive | Architect (Abbott) | **reactive** | Taqscreen MPX 2.0 | **reactive** | 1b | 1.6E+04 | neg*** | ST | nonreactive | **reactive** | **reactive** |
| 118(+215) | **reactive** |  | **reactive** | GFE Blut |  |  | 5,3E+01 | **pos***** |  |  |  |  |
| **119** | nonreactive | Architect (Abbott) | **reactive** | Taqscreen MPX 2.0 | **reactive** | 3a | 3.1E+04 | neg*** | ST | nonreactive | **reactive** | nonreactive |
| 119(+41) | **reactive** |  | **reactive** | GFE Blut |  |  | 3.2E+02 | ind*** |  |  |  |  |
| **120** | nonreactive | Vitros (Ortho) | **reactive** | Taqscreen MPX 2.0 | **reactive** | 1b | 1.1E+05 | neg*** | nonreactive | ST | nonreactive | nonreactive |
| 120(+28) | **reactive** |  | **reactive** | GFE Blut |  |  | 4.5E+05 | ind*** |  |  |  |  |
| **121** | nonreactive | Vitros (Ortho) | **reactive** | Taqscreen MPX 2.0 | nonreactive | 1a | 1.4E+02 | neg*** | nonreactive | ST | nonreactive | nonreactive |
| 121(+30) | nonreactive |  | **reactive** | GFE Blut |  |  | 8.8E+04 | neg*** |  |  |  |  |
| 121(+106) | **reactive** |  | **reactive** | GFE Blut |  |  | 1.1E+04 | **pos***** |  |  |  |  |
| **122** | nonreactive | Architect (Abbott) | **reactive** | Taqscreen MPX 2.0 | **reactive** | 4 | 2.0E+06 | neg*** | ST | nonreactive | **reactive** | **reactive** |
| 122(+92) | **reactive** |  | **reactive** | GFE Blut |  |  | 1.5E+05 | **pos***** |  |  |  |  |
| 122(+519) | **reactive** |  | **reactive** | GFE Blut |  |  | 6.7E+05 | **pos***** |  |  |  |  |
| **124** | nonreactive | Vitros (Ortho) | **reactive** | Taqscreen MPX 2.0 | **reactive** | 4a/4c/4d | 3.2E+05 | neg*** | nonreactive | ST | nonreactive | nonreactive |
| 124(+42) | **reactive** |  | **reactive** | GFE Blut |  |  | 5.3E+05 | ind*** |  |  |  |  |
| **125** | nonreactive | Architect (Abbott) | **reactive** | Taqscreen MPX 2.0 | **reactive** | 3a | 3.2E+06 | neg*** | ST | nonreactive | nonreactive | nonreactive |
| **126** | nonreactive | Vitros (Ortho) | **reactive** | Taqscreen MPX 2.0 | **reactive** | 1b | 2.5E+04 | neg*** | nonreactive | ST | nonreactive | nonreactive |
| 126(+14) | nonreactive |  | **reactive** | GFE Blut |  |  | 2.4E+04 |  |  |  |  |  |

ST – see the screening test result

*Chiron RIBA HCV 3.0 SIA Chiron; **HCV Blot 3.0 MP Diagnostics; ***recomLine HCV Mikrogen Diagnostik; ^ after antiviral treatment.

Table 2S. HCV NAT yields identification using different methods and formats.

| Method | format | Analytical sensitivity  [IU/ml] | period | donations | | Frequency of NAT yields  1/x and per 1 mln donations (95%CI) | |
| --- | --- | --- | --- | --- | --- | --- | --- |
|  |  |  |  | tested | positive |  |  |
| TMA  Procleix TMA HCV/HIV-1 | IDT | 6.2* | 2003-2004 | 287,569 | 3 | 1:95,856 | 10.4 (3.5-30.7) |
| TMA  Ultrio Plus | MP 8 | 24 | 2012-2014 | 490,509 | 1 | 1:490,509 | 2.0 (0.4-11.5) |
| TMA  Ultrio/Ultrio Plus/Ultrio Elite | IDT | 3 | 2005-2016 | 4,535,487 | 19 | 1:238,710 | 4.2 (2.7-6.5) |
| PCR  Cobas Amplicor | MP48 | 2,064 | 2000-2004 | 3,403,142 | 50 | 1:68,063 | 14.7 (11.1-19.4) |
| PCR  Cobas Ampliscreen | MP 24 | 504 | 2005-2007 | 1,554,270 | 14 | 1:111,019 | 9.0 (5.4-15.1) |
| R-T PCR  MPX / MPX 2.0 | MP 6 | 66 / 40.8 | 2007-2016 | 7,176,195 | 39 | 1:184,005 | 5.4 (4.0-7.4) |
| **In total** | **-** | **-** | **2000-2016** | **17,447,172** | **126** | **1:138,470** | **7.2 (6.1-8.6)** |

**Fig. S2** The frequency of HCV seronegative infections (number of NAT yields per 1 mln donations) in Blood Transfusion Centers, cumulated data for 2000-2016.
